# Application of chemotherapy combined with immunotherapy in first-line treatment of advanced esophageal cancer: fluorouracil or paclitaxel? – a meta-analysis

**DOI:** 10.3389/fonc.2025.1607715

**Published:** 2025-08-11

**Authors:** Xiaochen Chen, Huiping Yan, Hongyang Zhao, Pengfei Zhu

**Affiliations:** ^1^ Cancer Center, Department of Medical Oncology, Zhejiang Provincial People’s Hospital (Affiliated People’s Hospital), Hangzhou Medical College, Hangzhou, Zhejiang, China; ^2^ Department of Medical Oncology, Zhejiang Provincial People's Hospital Bijie Hospital, Bijie, Guizhou, China

**Keywords:** chemotherapy, immunotherapy, esophageal cancer, meta-analysis, paclitaxel, fluorouracil

## Abstract

**Introduction:**

Immunotherapy combined with chemotherapy is now the first-line standard for metastatic/recurrent esophageal cancer, with rapid immunotherapy advancements significantly improving patient outcomes. International guidelines recommend platinum-based combinations with either 5-fluorouracil or paclitaxel as first-line treatments. Currently, no head-to-head studies directly compare fluorouracil and paclitaxel regimens in esophageal cancer.

**Methods:**

We systematically searched online literature for clinical trials investigating first-line immunotherapy-chemotherapy combinations for esophageal cancer. Data were analyzed using Review Manager (RevMan) 5.3 software, with pooled results expressed as odds ratios (ORs) and 95% confidence intervals (CIs).

**Results:**

Both paclitaxel- and fluorouracil-based immunotherapy combinations demonstrated clinical benefits in overall survival, progression-free survival, and objective response rates. Notably, paclitaxel-based immunotherapy significantly increased ORR compared to fluorouracil-based regimens (OR 2.61 vs 1.58).

**Discussion:**

Our findings suggest that paclitaxel-immunotherapy combinations may offer enhanced disease control compared to fluorouracil-based approaches, warranting further studies to identify optimal patient populations and refine treatment strategies.

## Introduction

According to 2022 global cancer statistics, esophageal cancer accounted for 511,000 new cases (11th among all malignancies) and 445,000 deaths (7th) (1). In China, annual incidence reached 224,000 cases (7th) with 187,500 deaths (5th) (2). Most Chinese patients present with advanced-stage esophageal squamous cell carcinoma (ESCC), which carries a poor prognosis. For over three decades, chemotherapy has been the first-line standard for advanced esophageal cancer (3), though characterized by low response rates and high adverse events. Chemotherapy alone yields median overall survival under one year and a 5-year survival rate below 5%, demonstrating significant limitations. While targeted therapies have been explored, current approved agents are only indicated for esophageal adenocarcinoma (EAC) (4) and show limited efficacy against ESCC. Immunotherapy has emerged as a breakthrough, ushering in a new treatment era. Since 2017, immunotherapy has demonstrated excellent efficacy across solid tumors, with ESCC showing relatively favorable responses. Pivotal Phase III trials including KEYNOTE-590 (5), CheckMate 648 (6), RATIONALE 302 (7), RATIONALE-306 (8), GEMSTONE-304 (9) established that PD-1 inhibitors (monotherapy or chemotherapy-combined) achieve significant therapeutic benefits. Immune checkpoint inhibitors have progressively advanced from second-line to first-line ESCC treatment and from monotherapy to combinations, overcoming previous therapeutic bottlenecks. Current major guidelines recognize chemotherapy-immunotherapy combinations as the standard of care for ESCC, enabling personalized approaches for specific patient subgroups and significantly improving survival outcomes.

Most combination chemotherapy regimens incorporate single-agent drugs effective against esophageal cancer, with some regimens overlapping gastric cancer protocols. DCF (docetaxel/cisplatin/5-FU), ECF (epirubicin/cisplatin/5-FU), and cisplatin/5-FU are established backbone regimens for first-line ESCC treatment. Additional platinum-based combinations with taxanes, vinorelbine, gemcitabine, or irinotecan are alternative options. Notably, the latest Chinese Society of Clinical Oncology (CSCO) guidelines equally recommend platinum/fluorouracil (FC) or platinum/paclitaxel (TC) as category 1 options (10), whereas National Comprehensive Cancer Network (NCCN) guidelines prioritize FC (11). Although recent research focus has shifted toward combination therapies, chemotherapy regimen selection remains clinically significant, requiring consideration of toxicity profiles, treatment intervals, and patient tolerance. The RTOG 8501 trial established cisplatin/5-FU with definitive radiotherapy for unresectable locally advanced disease (12), but this regimen demonstrates substantial toxicity (42% grade 3 acute toxicity, 25% grade 3 late toxicity) and modest 5-year survival (26%).

In metastatic settings, paclitaxel has demonstrated efficacy, with preclinical evidence supporting its radiosensitizing properties. Despite lacking head-to-head comparisons, pathological complete response rates for paclitaxel-based regimens (19-53%) exceed those of standard cisplatin/5-FU. Consequently, paclitaxel-based concurrent chemoradiation is widely adopted clinically. Schnirer et al. at MD Anderson Cancer Center first evaluated paclitaxel-based chemoradiation in ESCC, noting favorable tolerability. The RTOG 0113 trial compared paclitaxel-based regimens against cisplatin/5-FU from RTOG 9405, showing comparable efficacy despite non-significant trends favoring paclitaxel (13). Similarly, the Chinese ESO-Shanghai Phase III trial found paclitaxel-based chemoradiation did not significantly prolong OS versus cisplatin/5-FU in locally advanced ESCC (14). Current evidence indicates comparable efficacy but distinct toxicity profiles between regimens, leaving regimen selection largely to clinician judgment based on individual patient factors.

This study analyzes Phase III RCTs investigating first-line immunotherapy-chemotherapy combinations for ESCC, specifically comparing efficacy differences between paclitaxel- and fluorouracil-based immunotherapy regimens to inform clinical decision-making.

## Methods

### Learning search strategy

This study systematically reviewed the literature according to Cochrane and PRISMA (the preferred reporting project for systematic review and meta-analysis) guidelines. Keywords included esophageal cancer, chemotherapy and immunotherapy. Literature databases covered English databases(PubMed, Web of Science, EMBASE, Cochrane Library) and Clinical trial registries (ClinicalTrials.gov, WHO ICTRP). Inclusion studies were designed for first-line treatment of esophageal cancer and included chemotherapy and immunotherapy combined with chemotherapy. Included studies were required to include one or more clinical outcomes: response rate, progression-free survival, and overall survival. The Jadad score was used to assess the quality of each study.

### Statistical analysis

Quantitative statistics were calculated using Review Manager (RevMan) software (Version 5.3; The Cochrane Collaboration, London, UK) and modeling was conducted using fixed effects or random effects. Heterogeneity was quantified using I2 statistics. All analyses were significant with p<0.05.

## Results

### The process and basic features of the included study

**Figure 1:**
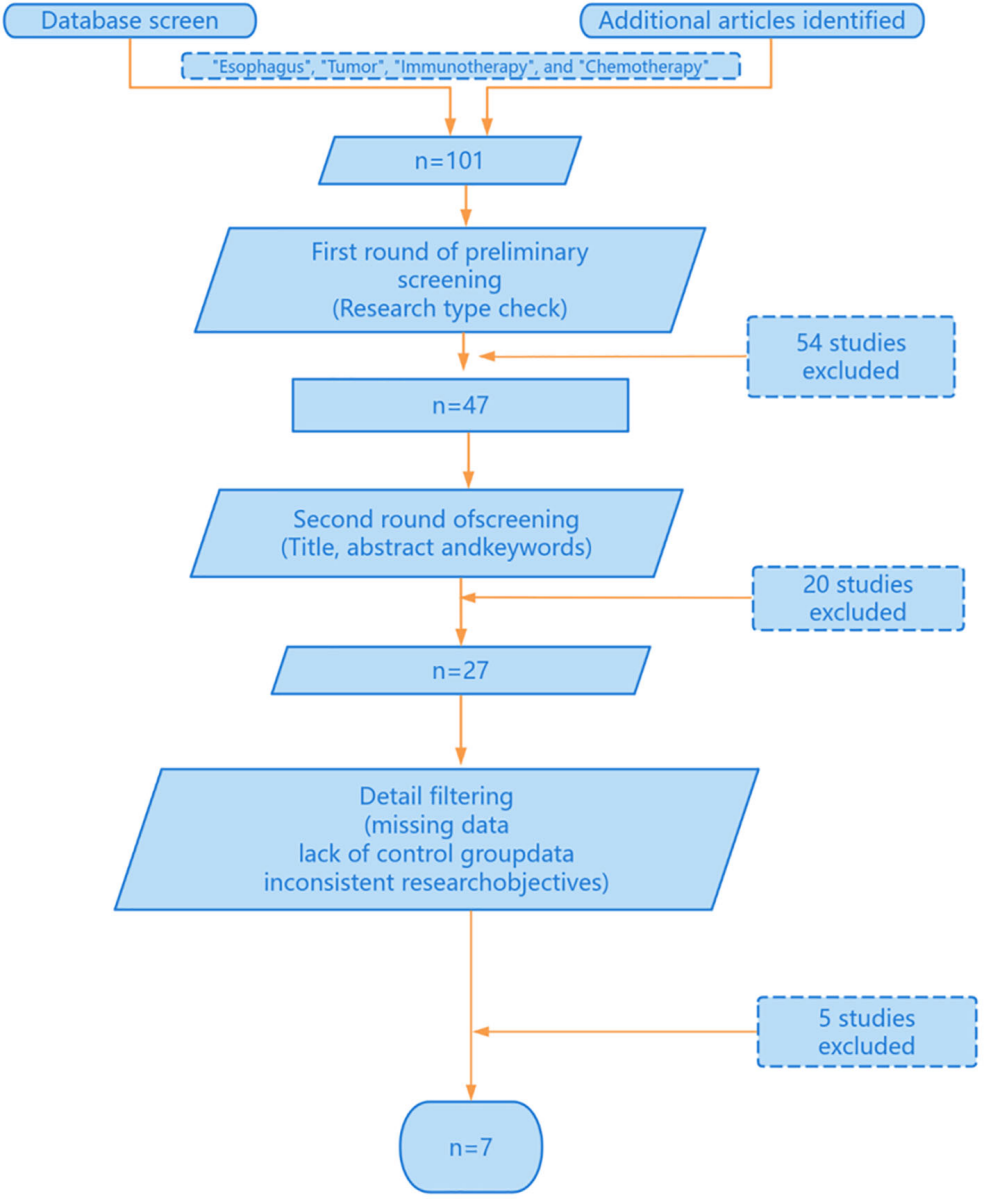
Research screening process for systematic review and meta-analysis.

This study was conducted through online database screening, and the flow chart is shown in **Figure 1**. Our systematic search covered database(PubMed, Web of Science, EMBASEand Cochrane Library) and Clinical trial registries(ClinicalTrials.gov, WHO ICTRP). According to the keywords “esophagus”, “tumor”, “immunotherapy” and “chemotherapy”, a total of 101 studies were identified in the database. 47 literatures were selected by reference type. Next, we conduct a second round of selection by title, abstract and keywords, and conduct a first reading of the full text if necessary and 20 studies were excluded. Next, 5 papers were excluded due to missing data, 7 papers were excluded due to lack of control group data, and 8 papers were excluded due to inconsistent research objectives. Finally, we identified seven randomized clinical studies reporting the efficacy and safety of first-line chemotherapy combined with immunotherapy in the first-line treatment of advanced esophageal cancer. The basic characteristics of the included studies are summarized in **Table 1**.

**Table 1:** Basic characteristics of the included study.

Clinical trial	Year	Study design	No. of patients	Included patients	Combination group	Control group	Reference(PMID)
Astrum-007	2022	RCT, phase 3 trial	551	ESCC	Serplulimab+FC	Placebo+FC	36732627
CM-648	2022	RCT, phase 3 trial	645	ESCC	Nivolumab+FC	FC	35108470
KN-590	2021	RCT, phase 3 trial	749	ESCC&EAC	Pembrolizumab+FC	Placebo+FC	34454674
ORIENT-15	2022	RCT, phase 3 trial	659	ESCC	Sintilimab+FC/TC	Placebo+FC/TC	35440464
Rationale-306	2023	RCT, phase 3 trial	649	ESCC	Tislelizumab+FC/TC	Placebo+FC/TC	37080222
Escort-1	2020	RCT, phase 3 trial	448	ESCC	Camrelizumab +Docetaxel	Docetaxel	32416073
JUPITER-06	2022	RCT, phase 3 trial		ESCC	Toripalimab+TC	Placebo+TC	35245446

ESCC, Esophageal squamous cell carcinoma; EAC, Esophageal adenocarcinoma; RCT, Randomized controlled trail; FC, cisplatin/oxaliplatin plus 5-fuorouracil; TC, cisplatin plus paclitaxel.

### Methodological quality

In this study, Jadad score was used to evaluate the quality of all included studies. All included studies showed acceptable quality (Jadad score greater than 4). One study received 7 stars, four received 6 stars, and two received 5 stars. Details of the assessment are shown in **Table 2**.

**Table 2:** The Jadad scale.

Clinical trails	Astrum-007	CM-648	KN-590	ORIENT-15	Rationale-306	Escort-1	JUPITER-06
Randomization (0-2)	2	2	2	2	2	2	2
Concealment of allocation (0-2)	2	1	2	2	2	2	2
Double blinding (0-2)	2	1	1	1	0	1	1
Withdrawals and dropouts (0-1)	1	1	1	1	1	1	1
Jadad score^a^	7	5	6	6	5	6	6

a Methodological quality of meditative movements studies reviewed using Jadad scoring criteria. Total score is 7. Score 1 to 3 considered as low quality; score 4 to 7 considered as high quality.

### Long-term survival of immunotherapy combined with chemotherapy versus chemotherapy alone

**Figure 2:**
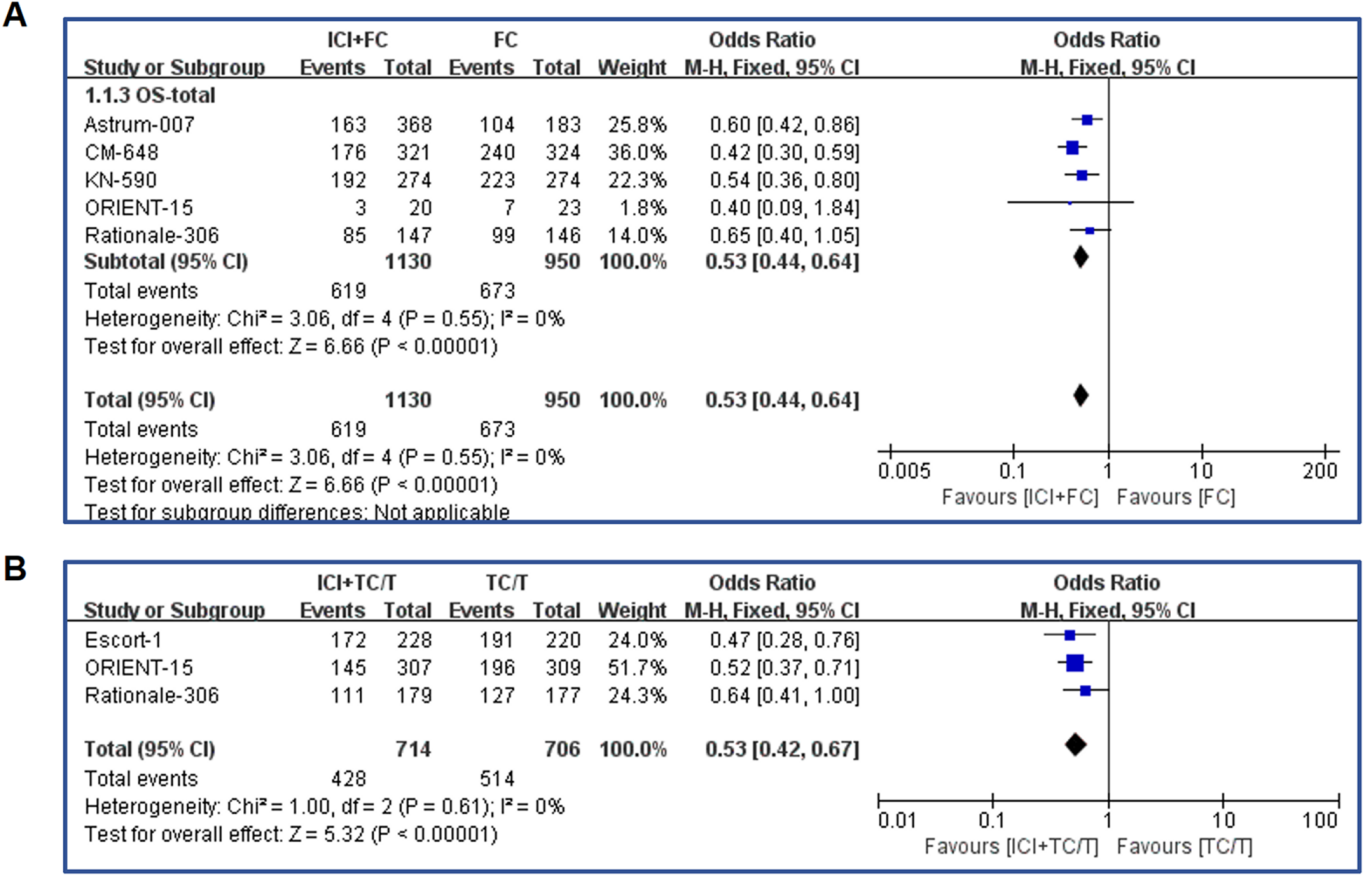
Forest plot of the overall survival. **(A)** Forest map analysis of literature on fluorouracil combined with immunotherapy; **(B)** Forest map analysis of literature on paclitaxel drugs combined with immunotherapy. ICI, Immune checkpoint inhibitors; FC, Fluorouracil combined with cisplatin/oxaliplatin; OS, Overall survival; TC, Taxol class drugs combined with cisplatin; T, Taxol class drugs. CIs, Horizontal lines represent 95% confidence intervals; M-H, Mantel-Haenszel; df, degrees of freedom.

We conducted a meta-analysis of all patients included in the literature, and the overall survival rate data analysis was shown in **Figure 2**. First, in patients treated with fluorouracil regimen, the combined immunotherapy group significantly extended overall survival compared with the control group (OR=0.53, 95% CI: 0.44-0.64, P<0.00001) (**Figure 2A**). Among patients treated with paclitaxel, the combination immunotherapy group also significantly extended overall survival compared to the control group (OR=0.53, 95% CI: 0.42-0.67, P<0.0001) (**Figure 2B**). In terms of odds ratio values, immunotherapy combined with fluorouracil chemotherapy appears to provide similar protection in overall survival as immunotherapy combined with paclitaxel (0.53 vs 0.53), reducing death risk by 47%.

### The comparison of the advantages of fluorouracil or paclitaxel chemotherapy regimen combined with immunotherapy in disease control

**Figure 3:**
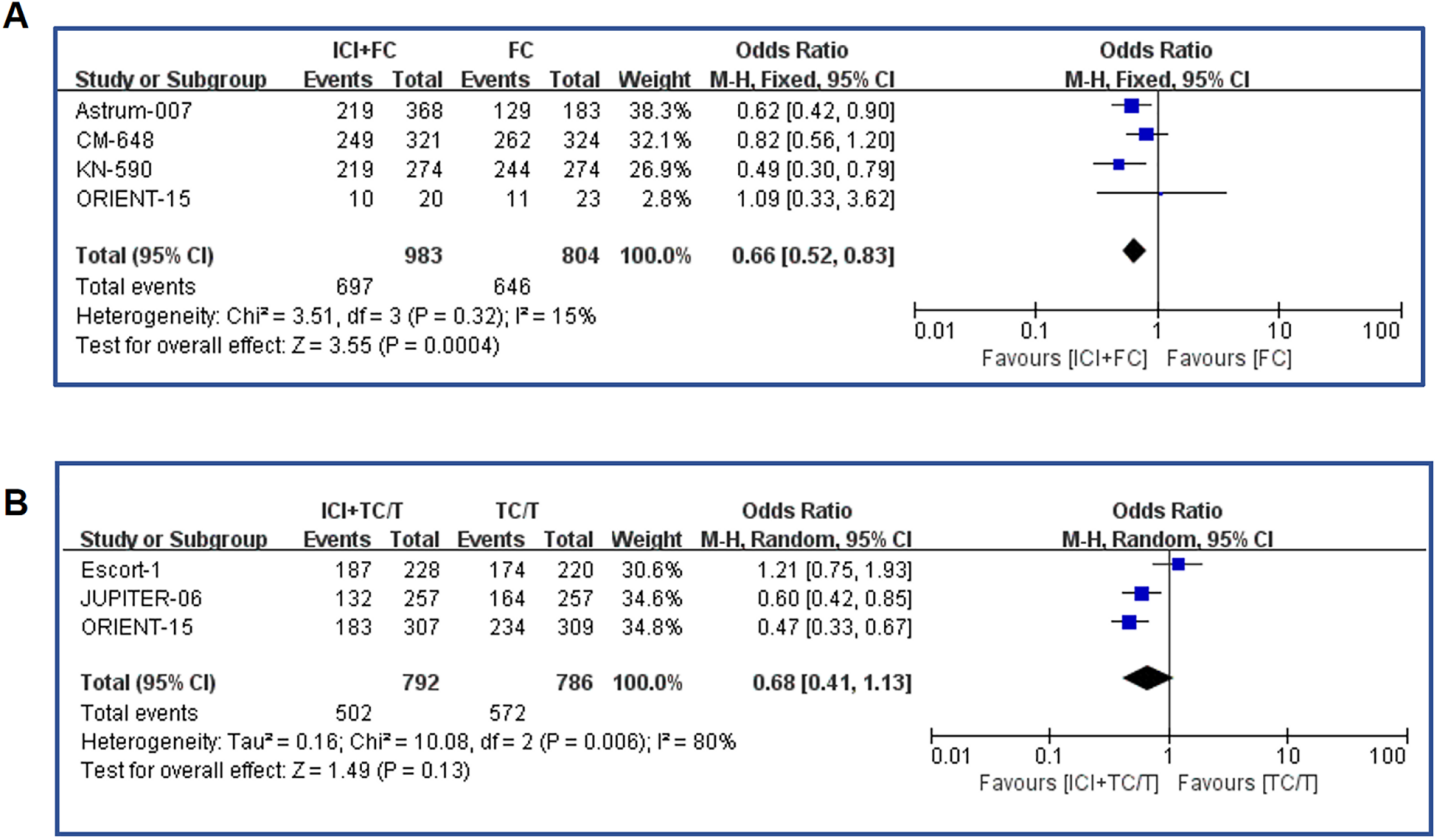
Forest plot of the progression-free survival. **(A)** Forest map analysis of literature on fluorouracil combined with immunotherapy; **(B)** Forest map analysis of literature on paclitaxel drugs combined with immunotherapy. ICI, Immune checkpoint inhibitors; FC, Fluorouracil combined with cisplatin/oxaliplatin; PFS, Progression-free survival; TC, Taxol class drugs combined with cisplatin; T, Taxol class drugs. CIs, Horizontal lines represent 95% confidence intervals; M-H, Mantel-Haenszel; df, degrees of freedom.

In our meta-analysis, we further collected the progression-free survival (PFS) of chemotherapy regimens in combination with immunotaxic drugs or fluorouracil versus chemotherapy alone. By summarizing all literature data on fluorouracil chemotherapy regimen combined with immunotherapy, the combination therapy significantly extended the PFS (OR=0.66, 95%CI: 0.53-0.83 P=0.0004)(**Figure 3A**). In the data summary of paclitaxel chemotherapy combined with immunotherapy, the combination therapy also significantly prolonged PFS (OR=0.64, 95%CI: 0.51-0.79 P<0.0001)(**Figure 3B**). In terms of odds ratio values, immunotherapy combined with fluorouracil chemotherapy appears to provide similar protection in PFS as immunotherapy combined with paclitaxel (0.64 vs 0.68), reducing death risk by about 30 percent.

**Figure 4:**
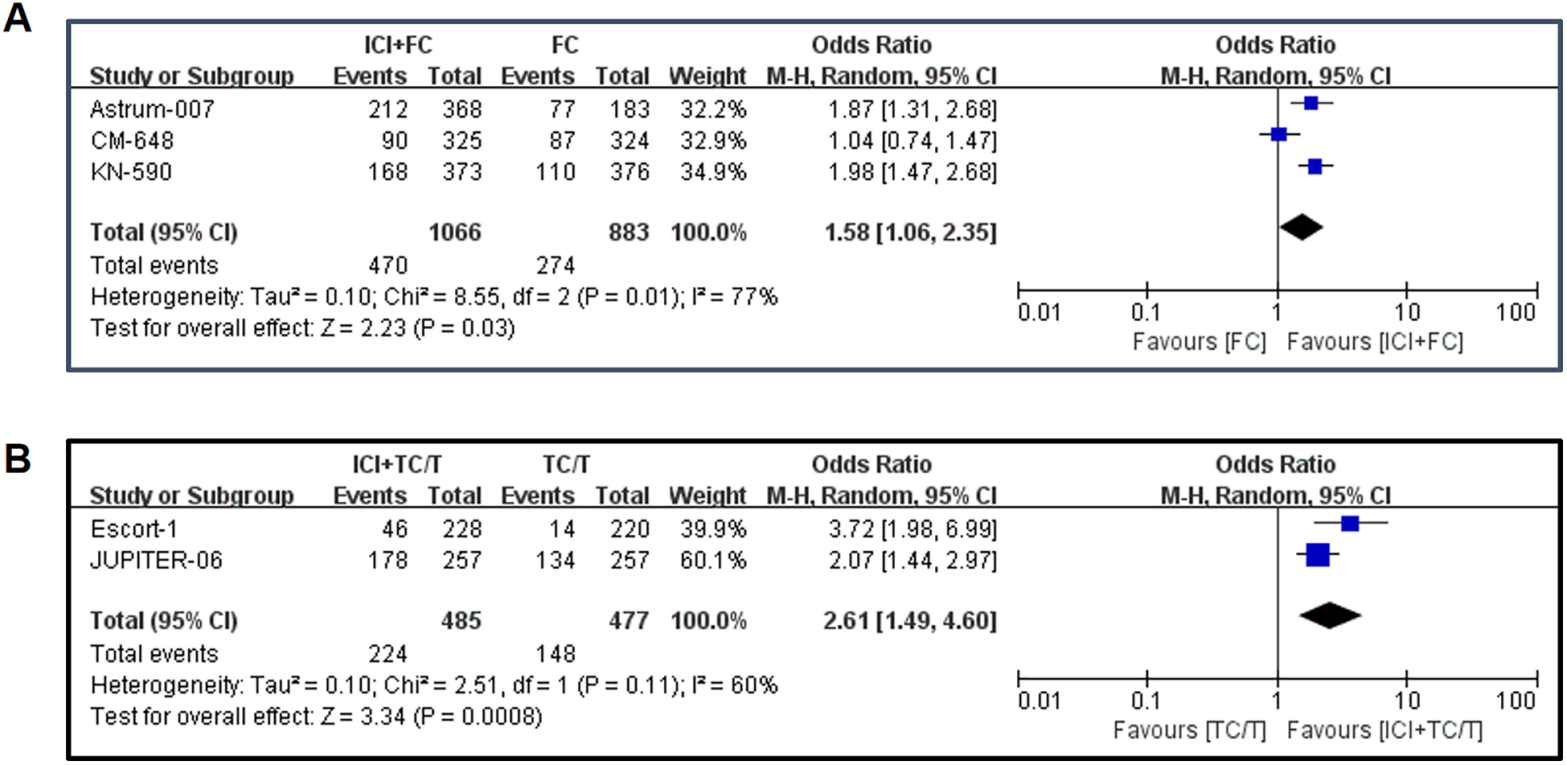
Forest plot of the objective response rates: **(A)** Objective response rates in fluorouracil combined with immunotherapy; **(B)** Objective response rates in paclitaxel drugs combined with immunotherapy. ICI, Immune checkpoint inhibitors; FC, Fluorouracil combined with cisplatin/oxaliplatin; ORR, Objective response rates; TC, Taxol class drugs combined with cisplatin; T, Taxol class drugs. CIs, Horizontal lines represent 95% confidence intervals; M-H, Mantel-Haenszel; df, degrees of freedom.

The study also had an endpoint called objective response rate (ORR), the percentage of patients whose cancer completely or partially shrinks or disappears after treatment. The meta-analysis of ORR was shown in **Figure 4**. The results showed that ORR was significantly increased in fluorouracil combined immunotherapy group compared with control group (OR= 1.58, 95%CI: 1.06-2.35, P=0.03)(**Figure 4A**). Similarly, paclitaxel combined with immunotherapy also resulted in ORR benefits (OR= 2.61, 95% CI: 1.49-4.60, P=0.0008)(**Figure 4B**). From the point of view of OR value, it seemed that paclitaxel combined immunization regimen could significantly improve ORR compared with fluorouracil combined immunotherapy.

### Publication bias

**Figure 5:**
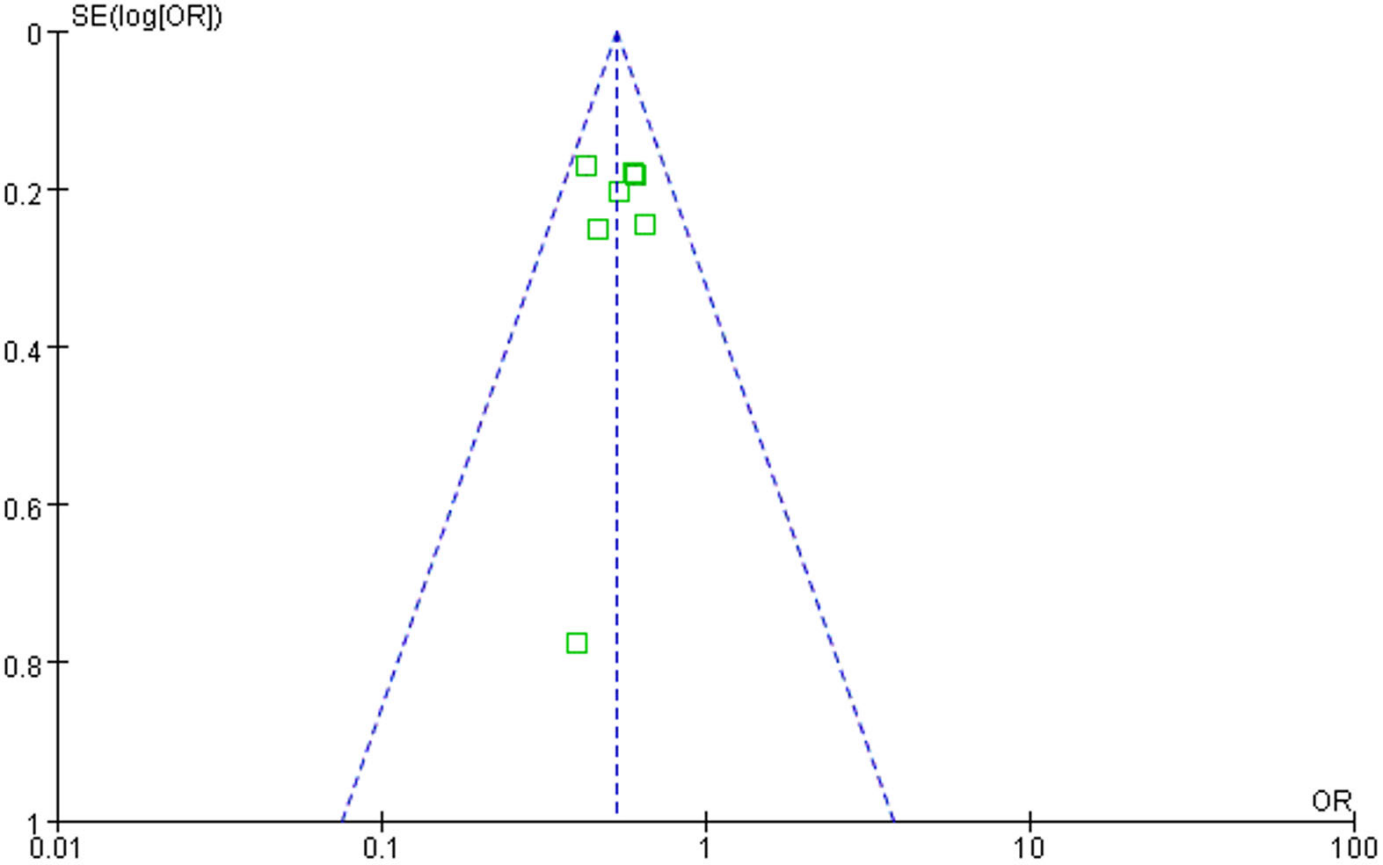
Funnel plot for publication bias.

Our study developed a funnel plot to detect publication bias (**Figure 5**). The visual funnel plot showed symmetry, indicating that there was no significant publication bias in this study.

## Discussion

Unlike traditional direct-comparison studies, this meta-analysis focuses on survival benefits of different chemotherapy regimens in the era of immunotherapy combination. Given subtle differences in chemotherapy recommendations across international guidelines (e.g., NCCN prioritizing fluorouracil-platinum while CSCO includes both paclitaxel/fluorouracil regimens) and the distinct pathological distribution in Chinese patients (squamous cell carcinoma >85%) versus Western populations (predominantly adenocarcinoma), our findings hold significant relevance for clinical practice in China.

The foundation for paclitaxel use in esophageal cancer dates back to a 2004 Phase I study (establishing platinum combination safety) and a 2007 Phase II trial (median OS reaching 12 months) (15). Notably, early comparative studies revealed toxicity profile differences: fluorouracil-based regimens showed significantly higher grade ≥3 adverse events than paclitaxel in neoadjuvant settings (16), and retrospective analyses of concurrent chemoradiation demonstrated lower hematological/non-hematological toxicities (grade ≥3: 19% vs 38%) and better treatment compliance with paclitaxel. This suggests paclitaxel may serve as an alternative for toxicity-vulnerable patients.

Although recent pivotal Phase III trials show no clear preference—with ESCORT-1 and JUPITER-06 selecting paclitaxel while ORIENT-15 and RATIONALE-306 adopted dual designs—our study reveals significantly higher ORR with paclitaxel-immunotherapy combinations (OR 2.61 vs 1.58). Potential mechanisms include: immunomodulatory effects through immunogenic cell death induction (17, 18) and MHC-I upregulation (19), radiosensitization properties via G2/M phase arrest (17, 20, 21), and metabolic advantages by avoiding fluorouracil’s DPD deficiency risks.

The ORR difference warrants cautious interpretation: despite paclitaxel’s superior response rates, the lack of significant OS/PFS differences suggests ORR may not reliably predict survival benefits. Toxicity profiles remain critical in regimen selection, particularly considering Chinese population characteristics. Paclitaxel requires careful use in diabetics due to dexamethasone premedication, while fluorouracil carries cardiac risks relevant for aging populations (22). China’s high diabetes prevalence (11.6%) amplifies paclitaxel’s corticosteroid concerns (23), and fluorouracil’s cardiotoxicity requires vigilant monitoring.

Against the backdrop of immunotherapy-chemotherapy becoming standard for advanced esophageal cancer, precision regimen selection demands individualized balancing. Paclitaxel offers potential immunotherapeutic synergy, superior toxicity management, and radiosensitization benefits. Current limitations include the absence of head-to-head survival data. Future research should explore biomarker-guided strategies: ERCC1/TUBB3 expression for paclitaxel sensitivity, PD-L1 CPS interaction analysis for immune synergy, and TDM2 polymorphism-based neurotoxicity prediction. We recommend Phase III trials directly comparing both regimens, focusing on identifying subgroups that may derive greater benefit from paclitaxel-immunotherapy combinations—particularly those eligible for sequential radiotherapy or with high PD-L1 expression.

## Data Availability

The original contributions presented in the study are included in the article/supplementary material. Further inquiries can be directed to the corresponding author.
